# Novel Single-Nucleotide Variants for Morpho-Physiological Traits Involved in Enhancing Drought Stress Tolerance in Barley

**DOI:** 10.3390/plants11223072

**Published:** 2022-11-13

**Authors:** Ibrahim S. Elbasyoni, Shamseldeen Eltaher, Sabah Morsy, Alsayed M. Mashaheet, Ahmed M. Abdallah, Heba G. Ali, Samah A. Mariey, P. Stephen Baenziger, Katherine Frels

**Affiliations:** 1 Crop Science Department, Faculty of Agriculture, Damanhour University, Damanhour 22516, Egypt; 2 Department of Agronomy and Horticulture, University of Nebraska-Lincoln, Lincoln, NE 68583, USA; 3 Department of Plant Biotechnology, Genetic Engineering and Biotechnology Research Institute (GEBRI), University of Sadat City (USC), Sadat City 32897, Egypt; 4 Plant Pathology Department, Faculty of Agriculture, Damanhour University, Damanhour 22516, Egypt; 5 Natural Resources and Agricultural Engineering Department, Faculty of Agriculture, Damanhour University, Damanhour 22516, Egypt; 6 Barley Research Department, Field Crops Research Institute, Agricultural Research Center, 9 Gamma Street-Giza, Cairo 12619, Egypt

**Keywords:** deficit irrigation, abiotic stress, marker-assisted selection (MAS), GWAS pleiotropic effect

## Abstract

Barley (*Hordeum vulgare* L.) thrives in the arid and semi-arid regions of the world; nevertheless, it suffers large grain yield losses due to drought stress. A panel of 426 lines of barley was evaluated in Egypt under deficit (DI) and full irrigation (FI) during the 2019 and 2020 growing seasons. Observations were recorded on the number of days to flowering (NDF), total chlorophyll content (CH), canopy temperature (CAN), grain filling duration (GFD), plant height (PH), and grain yield (Yield) under DI and FI. The lines were genotyped using the 9K Infinium iSelect single nucleotide polymorphisms (SNP) genotyping platform, which resulted in 6913 high-quality SNPs. In conjunction with the SNP markers, the phenotypic data were subjected to a genome-wide association scan (GWAS) using Bayesian-information and Linkage-disequilibrium Iteratively Nested Keyway (BLINK). The GWAS results indicated that 36 SNPs were significantly associated with the studied traits under DI and FI. Furthermore, eight markers were significant and common across DI and FI water regimes, while 14 markers were uniquely associated with the studied traits under DI. Under DI and FI, three (11_10326, 11_20042, and 11_20170) and five (11_20099, 11_10326, 11_20840, 12_30298, and 11_20605) markers, respectively, had pleiotropic effect on at least two traits. Among the significant markers, 24 were annotated to known barley genes. Most of these genes were involved in plant responses to environmental stimuli such as drought. Overall, nine of the significant markers were previously reported, and 27 markers might be considered novel. Several markers identified in this study could enable the prediction of barley accessions with optimal agronomic performance under DI and FI.

## 1. Introduction

Barley (*Hordeum vulgare* L.) is the fourth most important cereal crop worldwide [1]. Barley is an essential source of raw materials for the malting and brewing industries and is widely used as animal feed [2]. Barley grains can be used for human consumption in bread making, and are a rich source of fiber, vitamins, and minerals [3]. Barley is well known to be more drought tolerant than other cereal grains such as wheat (*Triticum* spp.) and corn (syn. maize, *Zea mays* L.) [4]. Despite its well-known drought stress tolerance, barley can suffer substantial grain yield losses, to the tune of 49–87%, due to drought stress [5], increasing the gap between production and consumption.

Additionally, the gap between cereal grains production and consumption is increasing because of the ongoing climate change and population and income growth, particularly in arid and semi-arid regions [6,7,8,9]. Furthermore, global warming is projected to increase the frequency of drought stress episodes, resulting in barley production losses [10,11]. Drought stress is the most devastating abiotic stress, and causes tremendous yield losses [12,13]. Areas in the world that are frequently affected by drought have dramatically increased, rising from 16.19% in 1902–1949 to 41.09% in 1950–2008 [14,15].

Drought stress tolerance is a complex quantitative trait controlled by many small-effect genes, and is often confounded by the growth stage [16]. The reproductive and grain filling stages are the most sensitive stages to drought stress [17]. Hence, enhancing barley tolerance to drought stress requires a profound understanding of the underlying genetic causes of the morphological and physiological changes observed in plants grown under drought stress [17]. In addition, it requires the induction of drought stress at the most appropriate time, in this case at flowering, when focusing on how drought stress affects reproduction. Variation among barley genotypes for drought tolerance exists, and has been well documented by several researchers [5,11,18,19,20,21,22,23,24]. These genetic variations among barley genotypes are expressed as morphological and physiological differences that can be used to identify drought-tolerant genotypes [5]. Diverse barley collections are publicly available and can be obtained from several gene banks worldwide [25]. As a result of the quantitative and complex mechanisms of drought tolerance, large and diverse collections of genotypes need to be exposed to water deficit in order to identify drought-tolerant genotypes [17]. The most critical step in evaluating any plant collection for drought tolerance is controlling the water supply to ensure exposing the materials being assessed to a genuine water shortage under field conditions at the appropriate time or developmental stage [17].

Field evaluation for drought stress tolerance is expensive, time-consuming, and highly affected by growth conditions [26]. Evaluation for drought stress tolerance under controlled environmental conditions, i.e., in a laboratory or greenhouse, is one approach proposed to reduce costs and improve assessment because of the ability to control water supply, i.e., avoiding unexpected rain [27]. However, controlled conditions might not genuinely represent the breeder’s targeted environmental conditions [27]. Thus, drought stress-tolerant genotypes identified under controlled conditions tend not to be as helpful as those identified under well-managed or controlled field studies [28]. To understand and work with the variability with field assays, plant breeders often implement DNA molecular markers to improve the selection accuracy by reducing genotype by environment interaction for complex traits such as drought tolerance [29,30].

The emergence and development of new DNA sequencing and high throughput genotyping technology has enabled the routine use of single nucleotide polymorphism (SNP) markers for marker-assisted selection (MAS) and gene pyramiding [31]. The single nucleotide polymorphism (SNP) platform generates thousands of SNPs using multiplexing scoring systems such as the Illumina GoldenGate and Infinium assays, which can be assayed in parallel, and software is available for automated allele scoring [32]. Thus, SNP has been found to be a valuable marker platform in GWAS studies across several crops [33].

Prior knowledge of the association between a marker and a trait is used in MAS to select for that trait in contemporary plant materials [34]. Therefore, the first step toward MAS is to identify markers linked with the desired traits, followed by validating these markers in several environments across several genetic backgrounds [35,36,37]. Phenotyping remains the cornerstone of plant breeding, as the first step in using molecular markers in plant breeding is to identify a marker–trait association, which requires precise phenotyping [29]. Furthermore, establishing accurate marker–trait associations requires a large number of lines in order to secure sufficient statistical power to detect a reliable marker–trait association [38].

Despite the attractiveness of MAS, the progress made in molecular plant breeding for quantitative traits such as drought remains limited [39,40]. This lack of progress can mainly be attributed to phenotyping, which has become the major bottleneck in molecular plant breeding applications, i.e., MAS [38]. Thus, in the current study, we focused on conducting extensive phenotyping for drought stress tolerance using a large collection of barley lines under drought-prone environments in Egypt. Egypt is one of the Mediterranean basin countries predicted to be adversely affected by climate change [41]. The predicted climate change and current periodic drought challenges impose an urgent need to enhance water use efficiency in barley in order to sustain acceptable grain production in arid and semi-arid regions such as Egypt. Thus, identifying SNP markers associated with drought stress tolerance in barley is expected to facilitate current efforts to improve water use efficiency in barley in Egypt.

In the present study, 426 elite barley lines from several US barley breeding programs were planted and evaluated in Egyptian field conditions under full irrigation (FI) and deficit irrigation (DI) to identify potential SNP markers associated with important traits such as the number of days to flowering (NDF), total chlorophyll content (CH), canopy temperature (CAN), grain filling duration (GFD), plant height (PH), and grain yield (Yield). The elite lines used in the current study have not previously been evaluated for drought stress. The results presented herein provide useful information on the importance of testing location and phenotyping for drought stress under drought-prone environments. After validating the novel markers discovered in this study under several environments and using different plant materials, these markers can be converted into Kompetitive allele-specific PCR (KASP) markers. KASP markers can be used in marker-assisted breeding schemes to accurately select allele donor parents or pyramid multiple alleles in the same genotype to enhance drought tolerance in barley. Therefore, novel markers associated with the traits identified in the current study are valuable for barley breeders in Egypt and other similar geographic regions.

## 2. Materials and Methods

### 2.1. Plant Materials and Testing Location

A panel of 426 spring barley lines and four commercially grown local check cultivars were investigated in this study. The barley lines were imported into Egypt from the barley breeding program at the University of Minnesota, MI, USA, in 2012. Further details regarding the lines’ description, pedigree, and the original development programs can be found on the T3/barley website (https://triticeaetoolbox.org/barley/, accessed on 2 October 2021) and in our previous publication [25]. Overall, the 426 imported barley lines contained two- and six-rowed lines, while the local check cultivars contained one two-rowed cultivar (Giza127) and three six-rowed cultivars (Giza132, Giza134, and Giza136). All genotypes were grown in Elkhazan during two consecutive growing seasons, 2018/2019 and 2019/2020, hereafter referred to as 2019 and 2020. Elkhazan is a commercial production farm located in the old Nile delta valley in northern Egypt (31°05′35.2″ N, 30°30′10.4″ E) with clay soil. The climate of the Elkhazan location is Dry–Mediterranean with a dry summer and mean annual rainfall ranging from 50–100 mm, with a maximum rainfall of 25 mm in January [17]. However, in several other regions (Southern Egypt) rainfall is very rare or close to zero. Therefore, using Egypt as a drought testing environment increases the relevance and magnitude of the findings to other countries in this region. Further climatic details on the study region are provided in Morsy et al. [17].

### 2.2. Agronomic Practices and Irrigation Treatments

For both two growing seasons, the preceding crop was corn. Surface soil samples (0–30 cm) were collected directly before planting and analyzed according to Klute et al. [42]. The main characteristics of the soil are presented in Table S1. Further details about the agro-ecological and climatic conditions in the testing location can be obtained from Morsy et al. [17].

During seedbed preparation, several steps were followed to minimize soil heterogeneity, specifically, disking to mix the previous crop’s residues with the sub-soil and laser leveling of the soil surface for improved distribution uniformity of the irrigation water. The recommended agronomic practices were applied in both growing seasons. Phosphorous fertilizer was applied during seedbed preparation at a rate of 75 Kg P_2_O_5_/ha in the form of superphosphate (15.5% P_2_O_5_). Potassium fertilizer was applied at 125 kg K_2_O/ha in the form of potassium sulfate (48% K_2_O equivalent) in two equal doses. The first dose was applied during seedbed preparation, while the second was applied after 30 days from sowing. Nitrogen fertilizer was used at the rate of 144 Kg N/ha in the form of ammonium nitrate (33.5%) in three doses; the first dose was applied at sowing, and the other two equal applications were at 21 and 45 days after sowing. During the booting growth stage, the plots were sprayed with TILT250 (Syngenta, Cairo, Egypt), which has Propiconazole as an active ingredient with a volume of 0.25% v.v., followed by Punch 40% EC (Orchem, Cairo, Egypt) at 0.185% v.v. after ten days from the TILT treatment to suppress disease development. For the first and second growing seasons, the sowing dates were 12 November 2018 and 16 November 2019, respectively. Chemical and manual weed control management were conducted to provide weed-free conditions across all trials.

Trials were subjected to drought stress by controlling irrigation, in which all genotypes were grown under 100% (full irrigation, FI) and 50% (deficit irrigation, DI) of the crop water requirements. All trials across growing seasons received the first irrigation directly after sowing to ensure uniform germination. Under FI, subsequent irrigations were applied after depleting 50% of the available water capacity (AWC). The AWC of the soil was calculated by subtracting water content at a permanent wilting point from water content at field capacity. When needed (50% of AWC), plots under FI were re-irrigated to 100% of the AWC in which the soil moisture content reached the field capacity. Irrigation intervals (days) for FI treatments were calculated by dividing the AWC (mm) by crop water requirements (mm/day). However, plants grown under DI received 50% of their water requirements by increasing the number of days between irrigations to double (compared to FI), resulting in a soil moisture content of ~30–35% of the AWC before re-irrigation. A water flow meter was installed in each trial to estimate the volume of the supplied water. The AWC and water requirements of barley were estimated according to Morsy et al. [17], in which soil moisture and weather parameters were considered.

### 2.3. Phenotypic Measurements

The number of days to flowering (NDF) was recorded visually as the number of days from sowing to anther exertion of 50% of the initial spikes. The total leaf chlorophyll content (CH) was estimated during the flowering stage using a spad-502 chlorophyll meter (spad-502 plus, Konica Minolta, Lincoln, NE, USA) on three randomly selected plants from each plot. The average of these three plants was used in the statistical analysis. Readings of CAN were measured during the flowering stage using a hand-held infrared thermometer (KM 843, Comark Ltd., Hertfordshire, UK). The CAN readings were taken from the same side of each plot at a one-meter distance from the edge and approximately 50 cm above the canopy at an angle of 30° to the horizontal. Readings were performed during the mid-day (between 1:00 and 3:00 pm) on sunny days. An average of five instantaneous CAN readings were recorded from each plot and used afterward in the analysis. The grain-filling duration (GFD, days) was measured as the period from flowering to physiological maturity. Plant height (PH, cm) was measured after physiological maturity on a random sample of five plants in each plot as the distance from the soil surface to the tip of the spike, excluding awns. Grain yield (Yield, ton/hectare) was measured by cutting all plants in each plot. After three days of air drying, plants were threshed using a locally made single plot thresher.

### 2.4. Genotypic Data

The lines used in the current study were genotyped using the 9K Infinium iSelect SNP genotyping array [43] from the USDA-ARS Biosciences Research Lab in Fargo, ND, USA. The single nucleotide polymorphism (SNP) markers were filtered by removing SNPs with missing values of > 10% and minor allele frequency (MAF) < 5%. Filtration resulted in 6913 high-quality SNPs, which were used afterward in the GWAS analysis. The iSelect_2013Consensus_AllSNPs linkage map was used to identify SNP marker positions. The linkage map and the SNP marker used in this study can be accessed through the T3 barley website: https://triticeaetoolbox.org/barley/downloads/downloads.php (accessed on 2 October 2021).

### 2.5. Experimental Design

The two water regimes (full and deficit irrigation) were assigned to the main plots, with two replicates within each trial. Genotypes were assigned to the subplots and arranged randomly into ten incomplete blocks of size 43. The size of the experimental unit (plot size) was four rows wide by 1.5 m long with 20 cm between rows within each replicate and growing season.

### 2.6. Statistical Analysis

A single environment analysis was conducted for the four trials (two years combined with two water regimes). Within each trial, analysis of variance, the approximated broad-sense heritability (sometimes known as repeatability [44]) and the coefficient of variation were estimated in order to characterize the quality of the measured traits within each trial. 

Moreover, the approximate broad-sense heritability (*H*) within trials was estimated as follows:

(1) H=σ2gσ2g+ σ2er

where $\sigma^{2}{}_{g}$ is the genotype variance (syn. Entry, which was treated as random), $\sigma^{2}{}_{e}$ is the residual variance, and r is the number of replicates within the trial. 

A multi-year mixed-effects analysis of variance model using SAS 9.2 (SAS v9.2; SAS Institute Inc., Cary, NC, USA) incorporating the two- and three-way interactions between growing seasons, genotypes, and water regimes for all phenotypic traits were implemented with the growing season, irrigation, genotypes, and genotypes × growing season, genotypes × irrigation, and genotypes × irrigation × growing season were tested as fixed effects. Moreover, the incomplete blocks nested within the complete blocks, growing season, and irrigation were treated as a random effect and used as an error term to test the significance of the growing seasons, irrigation, and irrigation × growing season [45]. The best linear unbiased estimates were obtained (BLUE) for each trial using the lsmeans statement in SAS (Supporting Information Table S2). 

Approximate broad sense heritability (*H*) across environments was estimated as follows:

(2) H=σ2gσ2g+σ2i/n+σ2enr

where $\sigma^{2}{}_{g}$ is the genotype variance, $\sigma^{2}{}_{i}$ is the variance of the genotype by environment interaction (syn. G × E), n is the number of environments, r is the number of replicates, and $\sigma^{2}{}_{e}$ is the residual variance [46].

### 2.7. Genome-Wide Association Mapping 

The estimated BLUEs for the phenotypic measurements obtained from each trial in conjunction with the SNP markers were subjected to a genome-wide association scan (GWAS). The GWAS scan was conducted using Bayesian-information and Linkage-disequilibrium Iteratively Nested Keyway (BLINK) implemented in GAPIT version 3 [47]. The first three principal components were derived from the SNP markers and used as covariate variables to account for the population structure (Q). Moreover, the kinship matrix (K-matrix) was obtained according to the VanRaden method [48] from the SNP markers among all pairs of lines; both Q and K were included in the GWAS model. Adjusted P-value following the Benjamini–Hochberg multiple testing procedure [49] was used to control the false discovery rate (FDR). Markers were declared significant in this study if they passed $-\log_{10}$ (for an adjusted *p*-value of 1 × 10^−5^) = 5. Estimaing the marker effect from the BLINK method was not applicable; thus, the markers effects were estimated by fitting the mixed effect model while simultanusely including both Q and K matrices.

### 2.8. Functional Annotation for the Significant Markers 

The significant markers detected from the GWAS scan were annotated using the IBSC_v2 barley genome assembly developed by the international barley sequencing consortium [50]. The flanking sequences 50 base pairs upstream and downstream of each significant SNP were used to conduct a BLAST search on the Ensemble Plants database [51]. The functions of the identified candidate genes were defined using the universal protein knowledgebase database (uniprot) https://www.uniprot.org/ [52]. Furthermore, the sequences of the significant markers were cross-referenced to the rice (*Oryza sativa* L.) and Arabidopsis (*Arabidopsis thaliana* (L.) Heynh.) genomes and their corresponding protein information.

## 3. Results

### 3.1. Phenotypic Performance within Environments

The mean performance, coefficient of variance, and broad sense heritability for all traits obtained with deficit (DI) and full irrigation (FI) for the two growing seasons, i.e., 2019 and 2020, were calculated in order to investigate the quality and suitability of the collected phenotypic data for genome-wide association analysis (GWAS, Table 1).

### 3.2. Number of Days to Flowering (NDF)

During the first growing season (2019), the NDF means were 86.8 and 90.92 days under deficit (DI) and full irrigation (FI), respectively. During the second growing season (2020), the NDF means were 83.30 and 87.32 days under DI and FI, respectively. The largest coefficient of variance (CV, 1.02%) for NDF was obtained from the FI regime in 2019, while the lowest CV (0.78%) was obtained from the FI in 2020. The broad-sense heritability (H^2^) for NDF under DI was 93 and 88% for the first and second growing seasons, respectively. Moreover, H^2^ for NDF under FI was 89% during the first and second growing seasons.

### 3.3. Canopy Temperature (CAN)

The mean CAN during (2019) was 31.57 and 26.32 °C for DI and FI, respectively. The mean CAN for the second growing season (2020) was 31.57 and 26.32 °C under DI and FI, respectively (Table 1). The largest CV (5.53%) for CAN was obtained from the DI during 2020, while the smallest CV (4.81%) was obtained from the FI during 2019. The broad-sense heritability (H^2^) for CAN under DI was 85 and 80% for the first and second growing seasons, respectively. Moreover, H^2^ for CAN under FI was 77 and 83% for the first and second growing seasons, respectively.

### 3.4. Total Chlorophyll Content (CH) 

During the 2019 and 2020 growing seasons, CH the means under DI were 31.57 and 29.22 SPAD units, respectively, while under FI, CH was 26.32 and 24.30 SPAD in 2019 and 2020, respectively. The CV for CH under DI was 5.96 and 8.55% for the first and second growing seasons, respectively. Moreover, the CV values for the FI were 5.28 and 8.92% in 2019 and 2020, respectively. The broad-sense heritability (H^2^) for CH under DI was 77% for the first and second growing seasons. Moreover, H^2^ for CH under FI was 87 and 84% for the first and second growing seasons, respectively.

### 3.5. Grain-Filling Duration (GFD) 

The mean GFD during (2019) was 26.27 for DI and 30.84 days for FI. In the second growing season (2020), the mean GFD was 26.34 under DI and 31.35 days under FI. The largest CV (5.43%) for GFD was obtained from DI in 2020, while the smallest CV (4.68%) was obtained from DI in 2019. The broad-sense heritability (H^2^) for GFD under DI was 92 and 86% for the first and second growing seasons, respectively. Moreover, H^2^ for GFD under FI was 90 and 88% for the first and second growing seasons, respectively.

### 3.6. Plant Height (PH)

In the 2019 and 2020 growing seasons under DI, the mean PH was 103.63 and 90.44 cm, respectively. Furthermore, the mean PH was109.43 and 96.20 cm under FI in 2019 and 2020, respectively. The CV for PH under DI was 2.94 and 4.26% for the first and second growing seasons, respectively. However, the CV values under FI conditions were 3.67% in 2019 and 3.34% in 2020. The H^2^ for PH under DI was 97 and 92% for the first and second growing seasons, respectively. Moreover, H^2^ for PH under FI was 95% for the first and second growing seasons.

### 3.7. Grain Yield (Yield)

The mean yields during 2019 were 3.40 and 4.27 tons/hectare for DI and FI, respectively. In the second growing season (2020), the mean yields were 3.12 and 3.85 tons/hectare under DI and FI, respectively. The largest CV (6.86%) for yield was obtained from DI during 2019, while the smallest CV (5.76%) was obtained from DI during 2020. The H^2^ for yield under DI was 91 and 87% for the first and second growing seasons, respectively.

### 3.8. Multiple Environments Analysis of Variance 

The analysis of variance for the total chlorophyll content, canopy temperature, grain filling duration, plant height, grain yield, and the number of days to flowering is presented in Table 2. According to the Bartlett test, variance across the six traits was homogenous for the two years and across the two stress treatments (DI and FI). Therefore, a combined analysis of variance was conducted. The combined analysis of variance (ANOVA) indicated a significant statistical effect (*p* < 0.01) for the Years (Y), Stress (DI and FI), and Genotypes (G) across all traits. Moreover, the combined ANOVA indicated a significant effect for the two- and three-way interactions across all traits. Among the four environments (two years and two water regimes), the highest heritability (94%) was observed for plant height, followed by GFD, Yield, and CH, which recorded heritabilities of 78, 73, and 72%, respectively. Furthermore, a moderate heritability (58%) was observed for NDF, while the smallest heritability (20%) was observed for CAN. 

### 3.9. Genome-Wide Association Scans (GWAS)

The combined analysis of variance indicated a substantial effect for genotypes by water stress interactions across the six traits. Thus, the estimated BLUEs for each trait obtained from DI (2019 and 2020) and FI (2019 and 2020) in conjunction with the SNP data were subjected to a genome-wide association scan (GWAS).

**Figure 1:**
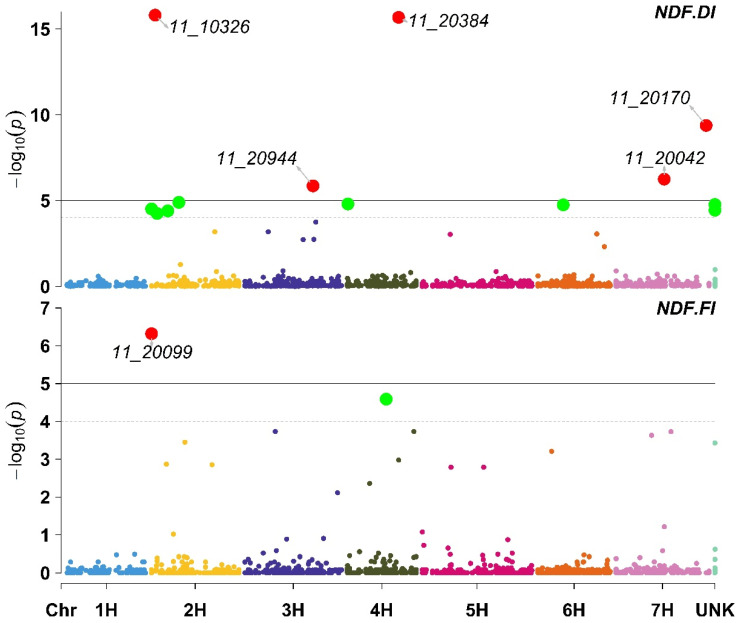
Manhattan plot displaying the results of the genome-wide association scan (red dots annotated dots refer to significant markers or markers with −log10 (p) more than 5, while the green dots refer to non-significant markers but have −log10 (p) between 4 and 5) for the number of days to flowering (NDF) using 426 barley lines under Deficit Irrigation (NDF.DI, upper plot) and Full Irrigation (NDF.FI, lower plot).

The results of the GWAS for NDF obtained from DI indicated that five SNP markers were significantly associated with NDF (Table S4). In contrast, only one SNP (11_20099) was significantly associated with NDF under FI (Figure 1). Additionally, under DI, the SNP marker 11_20099 was declared non-statistically significant because it had a corrected *p*-value of 3.22 × 10^−5^ (−log10 of 4.51), which is below the specified significance threshold. Moreover, all the significant SNP markers for DI and FI positively affected the NDF (Supporting Information Table S3).

**Figure 2:**
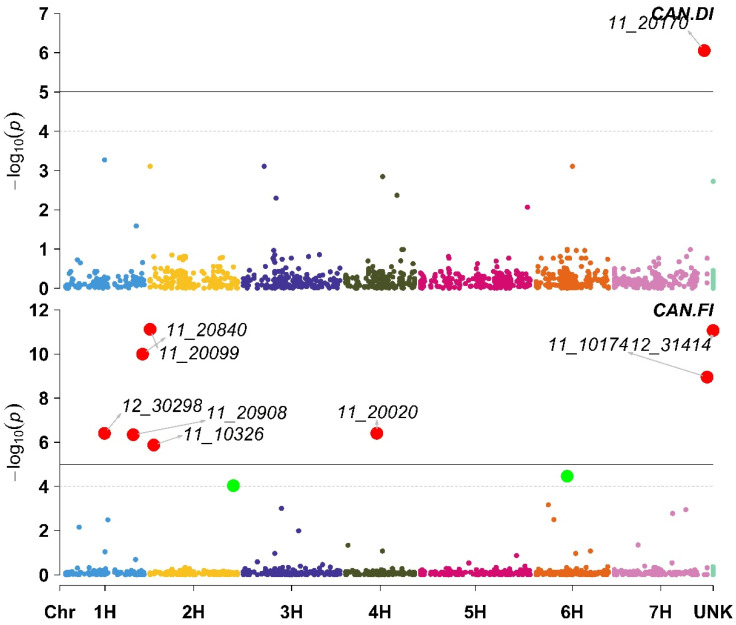
Manhattan plot displaying the results of the genome-wide association scan (red dots annotated dots refer to significant markers or markers with −log10 (p) more than 5, while the green dots refer to non-significant markers but have −log10 (p) between 4 and 5) for the Canopy Temperature (CAN) using 426 barley lines under Deficit Irrigation (CAN.DI, upper plot) and Full Irrigation (CAN.FI, lower plot).

A total of nine SNP markers were found to be significantly associated with CAN under DI and FI. Only one (11_20170 located on chromosome 7H) of these nine markers was significantly associated with CAN under DI. Furthermore, under FI, the associated markers were distributed among chromosomes 1H (11_20840, 12_30298, and 11_20908), 2H (11_20099 and 11_10326), 4H (11_20020), 7H (11_10174) and one marker (12_31414) was not mapped to a chromosomal location. (Figure 2). All markers that were associated with CAN under DI and FI had a positive effect on CAN, except for 11_20908, which was found to have a negative effect on CAN under FI (Supporting Information Table S3).

**Figure 3:**
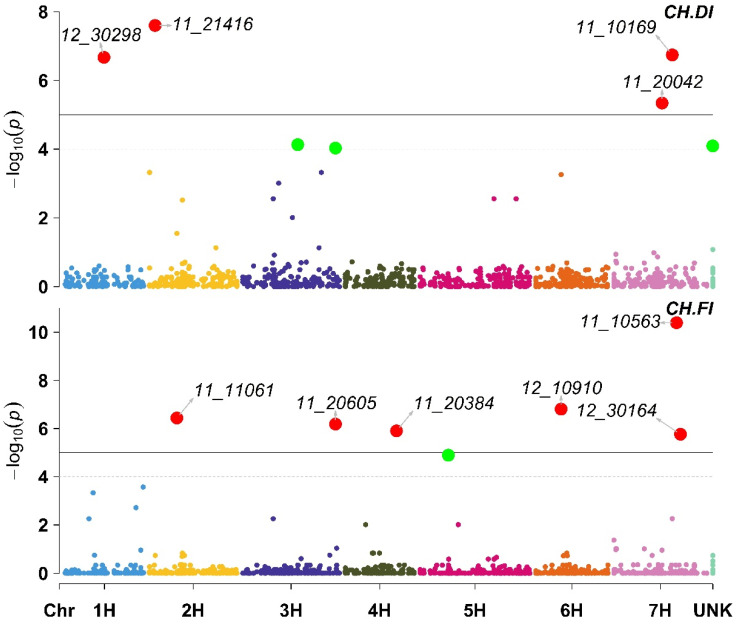
Manhattan plot displaying the results of the genome-wide association scan (red dots annotated dots refer to significant markers or markers with −log10 (p) more than 5, while the green dots refer to non-significant markers but have −log10 (p) between 4 and 5) for the total Chlorophyll Content (CH) using 426 barley lines under Deficit Irrigation (CH.DI, upper plot) and Full Irrigation (CH.FI, lower plot).

The GWAS results for the total Chlorophyll Content (CH) indicated that four markers were associated with CH under DI (Table S4). One of these markers (11_10169, located on chromosome 7 H) had a negative effect on CH, and the other three markers (11_21416 located on 2H, 12_30298 located on 1H, and 11_20042 located on 7H) had a positive impact on CH. Furthermore, under FI, six markers were associated with CH. One of these six markers (11_10563 located on chromosome 7 H) had a negative impact on the CH, while the other five markers (12_10910 on 6H, 11_11061 on 2H, 11_20605 on 3H, 11_20384 on 4H, and 12_30164 on 7H) had a positive impact on CH (Figure 3; Supporting Information Table S3).

**Figure 4:**
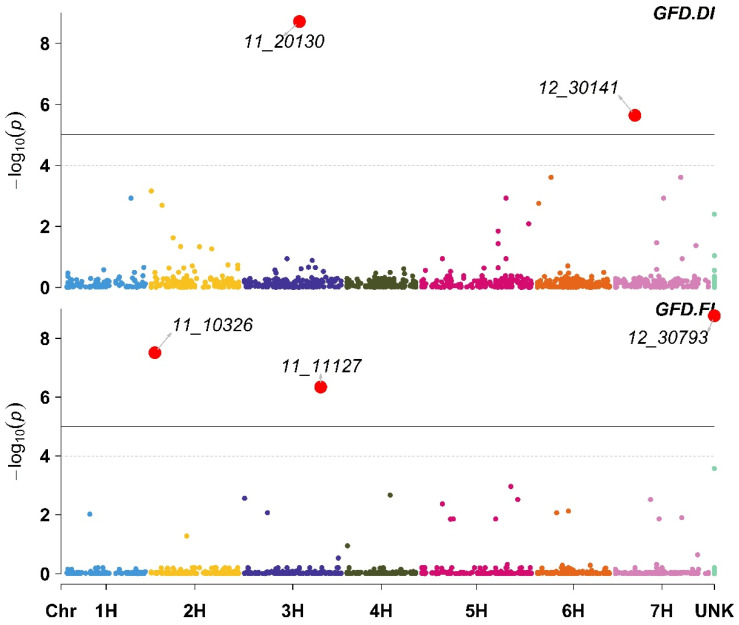
Manhattan plot displaying the results of the genome-wide association scan (red dots annotated dots refer to significant markers or markers with −log10 (p) more than 5, while the green dots refer to non-significant markers but have −log10 (p) between 4 and 5) for the Grain-Filling Duration (GFD) using 426 barley lines under Deficit Irrigation (GFD.DI, upper plot) and Full Irrigation (GFD.FI, lower plot).

Two markers were significantly associated with GFD under DI (Table S4). These two markers were located on chromosomes 3H (11_20130) and 7H (12_30141); both markers had a positive effect on GFD. Additionally, under FI, three markers were significantly associated with GFD; these three markers were located on chromosomes 2H (11_10326) and 3H (11_11127), while one marker (12_30793) was not mapped to a chromosomal location. Two of the three markers associated with FI had a positive effect on the GFD, while only one (11_11127) had a negative impact on the GFD (Figure 4; Supporting Information Table S3).

**Figure 5:**
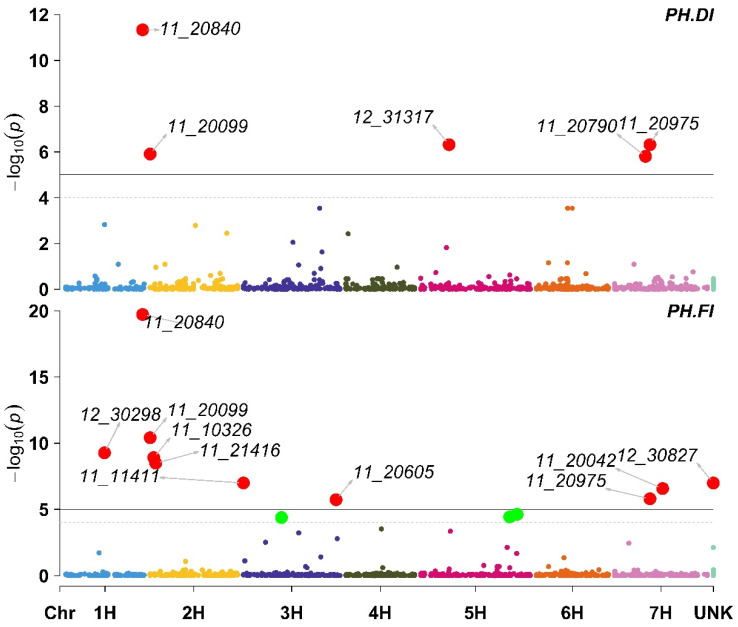
Manhattan plot displaying the results of the genome-wide association scan (red dots annotated dots refer to significant markers or markers with −log10 (p) more than 5, while the green dots refer to non-significant markers but have −log10 (p) between 4 and 5) for the plant height (PH) using 426 barley lines under Deficit Irrigation (PH.DI, upper plot) and Full Irrigation (PH.FI, lower plot).

Under DI, five markers were significantly associated with PH (Table S4). These five markers were located on chromosomes 1H (11_20840), 2H (11_20099), 5H (12_31317), and 7H (11_20975 and 11_20790). Among the five markers that were associated with plant height, only one marker (11_20975) was associated with increased plant height. In contrast, the other four were associated with a reduction in plant height. Under FI, nine markers were significantly associated with plant height. The nine markers were located on chromosomes 1H (11_20840 and 12_30298), 2 H (11_20099, 11_10326, and 11_21416), 3H (11_11411), 7H (11_20042 and 11_20975), and one marker with no available mapping information (12_30827). Two markers (11_11411 and 11_20975) were associated with a reduction in plant height, while the other seven markers were associated with increased plant height (Figure 5; Supporting Information Table S3).

**Figure 6:**
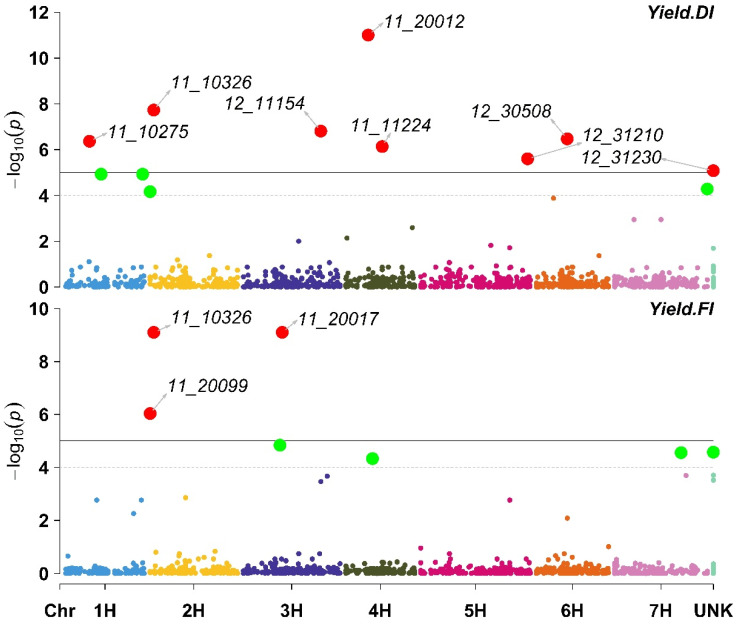
Manhattan plot displaying the results of the genome-wide association scan (red dots annotated dots refer to significant markers or markers with −log10 (p) more than 5, while the green dots refer to non-significant markers but have −log10 (p) between 4 and 5) for grain yield (Yield) using 426 barley lines under Deficit Irrigation (Yield. DI, upper plot) and Full Irrigation (Yield. FI, lower plot).

The GWAS results for grain yield under DI indicated that eight markers were associated with grain yield (Table S4). The eight markers were distributed among several chromosomes, i.e., 1H (11_10275), 2H (11_10326), 3H (12_11154), 4H (11_11224), 5H (12_31210), 6H (12_30508), and one marker (12_31230) that was not mapped to a chromosomal location. Among the eight markers, only 11_11224 was associated with a reduction in grain yield. Moreover, three markers (11_10326, 11_20099, and 11_20017) were significantly associated with grain yield under FI. Among the three markers, only 11_20017 was associated with a reduction in grain yield (Figure 6; Supporting Information Table S3).

### 3.10. Significant Pleiotropic Effect Markers 

**Figure 7:**
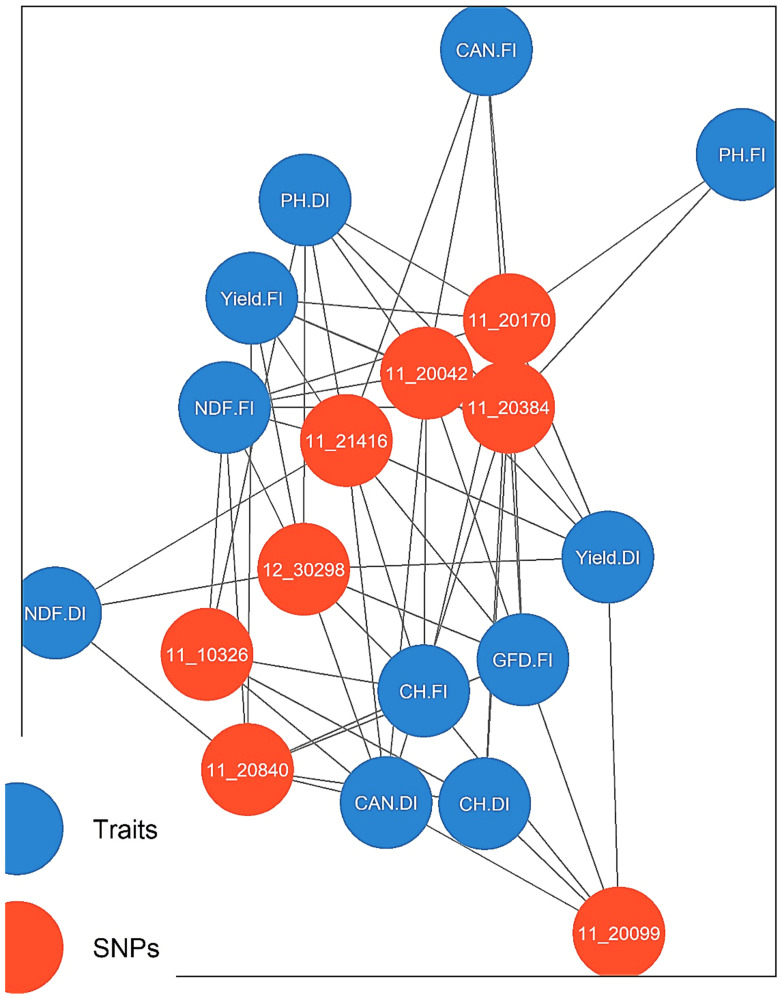
Summary of the genome-wide association scans for the significant pleiotropic effect markers associated with the Number of Days to Flowering (NDF), Canopy Temperature (CAN), Total Chlorophyll Content (CH), Grain-Filling Duration (GFD), Plant Height (PH), and Grain Yield (Yield) in 2019 and 2020 under Full Irrigation (FI) and Deficit Irrigation (DI).

The GWAS results revealed that eight SNP markers are significantly associated with at least two phenotypic traits (Figure 7), suggesting the possible presence of pleiotropic or indirect effects of these markers on barley phenology under DI and FI. Among these SNP markers is 12_30298, located on chromosome 1H, which was found to be significantly associated with CAN under FI and CH under DI. Moreover, 11_20840 is another SNP marker located on chromosome 1H, and was found to be significantly associated with CAN under FI, PH under FI and DI, and yield under DI. Furthermore, three markers located on chromosome 2H, i.e., 11_20099, 11_10326, and 11_21416, were found to be associated with at least three phenotypic traits. More specifically, 11_20099 was significantly associated with NDF, PH, and Yield under FI and DI, while it was associated with CAN under FI.

Additionally, 11_10326 was found to be significantly associated with NDF under DI; CAN, GFD, and PH under FI and yield under both DI and FI. The third marker on chromosome 2H with pleiotropic effect is 11_21416, which was found to be associated with NDF under DI and FI, CH under DI, and PH under FI. One marker on chromosome 4H was significantly associated with NDF under DI and CH under FI. On chromosome 7H, two markers (11_20042 and 11_20170) with pleiotropic effect under DI were identified; 11_20042 was found to be significantly associated with NDF and CH, while 11_20170 was significantly associated with NDF and CAN.

## 4. Discussion

In the current study, 426 barley genotypes were evaluated under two water regimes DI and FI. Our results indicated a significant interaction between genotypes and water regime, implying a different pattern of response among the genotypes under DI compared to FI. Because the interaction between genotypes and water regime was significant, a separate GWAS scan was conducted for the studied traits under DI and FI in order to identify potential SNP markers uniquely associated with the studied traits under each water regime.

The genotypes and SNP markers used in the current study were previously investigated to identify QTLs associated with phenotypic performance under normal growth conditions in the USA [53]. Furthermore, the results of the previous investigations indicated that three principal components (PCA) were sufficient to account for the population structure [25]. However, the panel used in the current study has never been evaluated for drought stress. The main objective of this study was to use GWAS to identify potential genomic regions contributing to enhanced barley tolerance to drought stress. 

One of the essential characteristics behind barley’s tolerance to drought stress is NDF. Genotypes associated with early flowering allow plants to complete pollination and grain development in a shorter period [54]. Thus, early flowering plants are usually use water efficiently and avoid drought stress and the subsequent indirect heat stress at the plant’s most critical growth stages, i.e., flowering. Our GWAS model focused on identifying markers associated with NDF under DI and FI in this study. The GWAS model successfully identified five SNP markers associated with NDF under DI. Two of these five markers were annotated to two genes, i.e., 11_20170 and 11_20944 to genes *HORVU7Hr1G121870.1* and *HORVU3Hr1G089580*, respectively. The gene *HORVU7Hr1G121870.1* was found to play a role in repressing flowering and promoting defense gene expression and synthesis of defensive secondary metabolites in both barley and *A. thaliana* [55]. The gene *HORVU3Hr1G089580* encodes Ribonuclease II. Ribonuclease is involved in the flowering and vegetative stages of development in other plants [56]. However, we could not find previously published reports linking ribonuclease II to flowering in barley. Three other markers that were found to be significantly associated with NDF under DI did not have known gene annotations (11_10326, 11_20042, and 11_20384). The annotation results of the marker 11_10326, aka POPA1_0326, indicated that the marker sequence is involved in encoding a membrane-related protein (CP5). The CP5 protein was found to be related to post-flowering drought tolerance in barley [57].

The annotation of the marker 11_20042 indicated that it is involved in encoding the protein histone deacetylase 11. Histone deacetylase is involved in plant responses to abiotic stress [58]. The third marker, 11_20384, encodes protein glycine-rich cell wall structural protein2 precursors, which play a potential role in abscisic acid, stress, and ripening-induced gene (ASR) in barley [59].

One marker (11_20099) was significantly associated with NDF under both FI and under DI (*p* < 1 × 10^−5^). In a previous publication, the marker 11_20099 was discovered to be associated with polyphenol oxidase activity and environmental adaptation in barley [60,61].

Canopy temperature (CAN) is another vital trait that is essential in drought stress tolerance [62]. Under DI, one marker (11_20170) was significantly associated with CAN, which in this study was found to be associated with NDF under FI as well. Moreover, under FI, eight SNP markers were associated with CAN. The first marker was 11_10174, which was annotated to the *HORVU7Hr1G121700* gene, which encodes catalase 2 (CAT2). Catalase 2 (CAT2) activity increases in leaves experiencing water deficits and H_2_O_2_ accumulation [63].

The SNP marker 11_20020, which was found to be associated with CAN, was previously reported by [64]; they indicated an association between that marker and barley response to abiotic stresses (i.e., drought, salinity, and heat stress). The SNP marker 11_20020 was annotated to *HORVU4Hr1G058560* gene, which encodes plant-specific domain TIGR01589 family protein.

Marker 11_10326 was associated with CAN under FI and NDF under DI, and was previously reported to be associated with shoot dry weight and beta-glucan [64]. Another marker, 11_20099, that was found to be associated with CAN was previously reported and was found to be associated with NDF under FI in this study. Additionally, the marker 11_20840 that was found to be associated with CAN was annotated to *HORVU1Hr1G094480,* which encodes protein endopeptidase Clp. The protein endopeptidase Clp plays an essential role in chloroplast and protein degradation in senescing leaves [65]. As part of their “escape” mechanism, plants reduce the size of their canopy in reaction to stress by accelerating senescence and leaf abscission. Despite the fact that an accelerated plant senescence strategy assists in the next generation’s survival (i.e., seed production) under stress, annual plant species suffer significant losses in agricultural yield, which result in a concurrent decrease in production and substantial financial losses to farmers.

Marker 11_20908 was associated with CAN under FI and was annotated to the *HORVU1Hr1G086110* gene, which encodes S-like ribonuclease. Marker 12_30298 was associated with CAN under FI; the annotation of this marker did not return any known barley genes. Marker 12_31414 was associated with CAN in this study and was annotated to the *HORVU4Hr1G014490* gene.

It is widely known that drought stress reduced the total chlorophyll content (CH) of the flag leaves [66]. In total, ten markers were found to be associated with CH under FI and DI. Four markers (11_10169, 11_21416, 12_30298, and 11_20042) were associated with CH under DI. Marker 11_10169 was annotated to the *HORVU7Hr1G098440* gene, which encodes a cell wall-modifying enzyme rapidly upregulated in response to environmental stimuli such as drought [67]. Marker 11_21416 has no available annotation information, while marker 12_30298 was previously reported in this study, in which it was found to be significantly associated with CAN under FI. Moreover, marker 11_20042 was reported before, and was found to be associated with NDF in the current study.

Six markers (11_10563, 11_11061, 11_20384, 11_20605, 12_10910, and 12_30164) were associated with CH under FI. Markers 11_10563 and 11_20605 have no available annotation information. Marker 11_11061 was annotated to the *HORVU2Hr1G023590* gene, which encodes B-S glucosidase 44. Marker 11_20384 was previously reported and found to be associated with NDF under DI. Marker 12_10910 was annotated to the *HORVU6Hr1G021170* gene and was previously reported by Igartua et al. [68] to be associated with plant height. Marker 12_30164 was annotated to the *HORVU7Hr1G105460* gene and was previously reported by Muñoz-Amatriaín et al. [64], in which they indicated a significant association between this marker and arabinoxylan content, a major component of cell walls.

Grain-Filling Duration (GFD) is another trait found to be adversely affected by drought stress, with drought causing a reduction in GFD [69]. Under DI, two markers (11_20130 and 12_30141) were associated with GFD. Even though marker 11_20130 was not annotated to known genes, its sequence was aligned to the genomic region involved in encoding photosystem II 22 kDa protein and a chloroplast precursor. Marker 12_30141 was annotated to the *HORVU7Hr1G021840* gene, which encodes cysteine synthase protein. The cysteine synthase protein is involved in drought tolerance in plants [70].

Under FI, three markers (11_10326, 11_11127, and 12_30793) were associated with GFD. Marker 11_10326 was reported in this study before and found to be associated with CAN. Marker 11_11127 was annotated to the *HORVU3Hr1G096210* gene, which encodes secondary cell wall-related glycosyltransferase family 47 protein. Marker 12_30793 was not annotated to known barley genes.

Drought stress reduces plant height (PH) by reducing cell-division enlargement and differentiation [71]. Five markers (11_20840, 11_20975, 12_31317, 11_20099, and 11_20790) were significantly associated with PH under DI. Among these five markers, 11_20840 was previously reported in this study and was associated with CAN under FI. Marker 11_20975 was not annotated to any known genes in barley. However, the marker 11_20975 sequence was annotated to the barley genomic region involved in encoding FIP1 protein. FIP1 is critical for plant development and root responses to abiotic stresses [72]. Marker 12_31317 was annotated to the *HORVU5Hr1G038720* gene, which encodes Eukaryotic translation initiation factor 2B (eIF-2B) family protein. Marker 11_20099 was found to be associated with CAN under FI and NDF under FI and DI. Marker 11_20790 was annotated to the *HORVU3Hr1G034290* gene and was previously reported by Borràs-Gelonch and Romagosa [73], who found it to be associated with peduncle length in barley.

Under FI, ten markers were associated with PH; three of these ten markers (11_20840, 11_20099, and 11_20975) were significantly associated with PH under DI. Moreover, among the significant markers for PH under FI, four markers (11_10326, 11_20042, 11_21416, 12_30298, and 11_20605) were previously reported and found to be significantly associated with other traits in this study. Only two markers (11_11411 and 12_30827) were uniquely associated with PH under FI. Marker 11_11411 was annotated to the *HORVU3Hr1G111600* gene, which encodes a protein involved in plant immunity (ILITYHIA); this gene has a pleiotropic effect on plant size, with mutant plants tending to be smaller in size [74].

There have been several reports on the negative impact of drought stress on barley grain yield (Yield) production [5]. Under DI, eight markers (11_20012, 11_10326, 12_11154, 12_30508, 11_10275, 11_11224, 12_31210, and 12_31230) were associated with yield. Marker 11_20012 was annotated to *HORVU4Hr1G010700.1,* which encodes protein 50S ribosomal L12-2, a chloroplast precursor. Marker 11_10326 has unknown annotation information; however, an association between this marker and shoot dry weight and beta-glucan in barley was previously reported [64]. Marker 12_11154 was annotated to the *HORVU3Hr1G096830* gene, which encodes serine carboxypeptidase-like protein (SCPL). SCPL plays a vital role in stress response, growth, development, and pathogen defense [75]. Marker 12_30508 was annotated to the *HORVU6Hr1G053760* gene, which encodes a drought-responsive microRNA (FUS3-complementing gene2). That microRNA was previously identified in *Sorghum bicolor* (L.) and found to be responsive to drought [76]. Marker 11_10275 was annotated to the gene *HORVU1Hr1G013680*; this marker was reported in other studies, in which it was found to be significantly associated with grain yield [77]. Marker 11_11224 was annotated to the *HORVU4Hr1G067520* gene, which encodes glycosyl hydrolases family 31 protein (GH31). GH31 is a diverse group with a range of different Carbohydrate-Active EnZymes (CAZy). In response to drought stress, GHs catalyse the hydrolysis of O- or S- glycosidic bonds to release sugars and provide the plant with energy for development.

Marker 12_31210 was annotated to the gene *HORVU5Hr1G124350*, which encodes the DANA2 protein. Marker 12_31210 was previously reported and found to be associated with seed dormancy in barley [78]. Marker 12_31230 was annotated to the gene *HORVU3Hr1G002800*, which encodes DEA(D/H)-box RNA helicase family protein. This protein family is involved in abiotic stress tolerance in plants [79].

Additionally, three markers (11_10326, 11_20017, and 11_20099) were associated with yield under FI. Marker 11_10326 was significantly associated with yield under DI, while 11_20099 was significantly associated with other traits in this study (NDF, CAN, PH, and Yield). Marker 11_20017 was uniquely significant with respect to yield under FI. This marker was not annotated to a specific barley gene. However, it was reported by other researchers and found to be associated with yield in barley [79].

The plant’s overall performance under drought stress is influenced by the relationships among several morphological and physiological traits [80]. In the current study, we focused on six morpho-physiological traits (NDF, CAN, CH, GFD, PH, and Yield) that were highly influenced by drought stress conditions [81]. Thus, we expected to identify markers with pleiotropic effects on these traits. Overall, under DI, three SNP markers (11_10326, 11_20042, and 11_20170) had a pleiotropic effect on at least two traits. Marker 11_10326 was significantly associated with NDF and yield. Marker 11_20042 was significantly associated with NDF and CH, while marker 11_20170 was significantly associated with NDF and CAN.

Additionally, under FI, five markers (11_20099, 11_10326, 11_20840, 12_30298, and 11_20605) had pleiotropic effects on at least two traits. Interestingly, marker 11_20099 was significantly associated with NDF, CAN, PH, and yield, while 11_10326 was significantly associated with NDF, GFD, PH, and Yield. Moreover, marker 11_20840 was significantly associated with CAN, PH, and Yield, while 12_30298 was significantly associated with CAN and PH. Finally, marker 11_20605 was significantly associated with CH and PH. The pleiotropic effect of several of the significant markers detected in this study under DI and FI was expected, as the traits that we used are among the traits that play a key role in barley grain yield in both drought-stressed and favorable conditions. It is known that grain yield is the cumulative effect of many traits and that drought stress tends to reduce, NDF, CH, GFD, PH, and Yield while increasing CAN [78,82].

The interrelationships among NDF, CAN, CH, GFD, PH, and grain yield might explain the pleiotropic role of certain loci or markers in these traits. Several researchers have extensively studied the contributory relationship between NDF and grain yield [82,83]. They have reported that exposing plants to drought during the flowering stage causes substantial losses in grain yield [82,84]. Thus, pleiotropic roles for flowering-related loci in CAN, CH, GFD, PH, and grain yield was expected in this study, as this has been previously reported [85,86].

## 5. Conclusions

This study identified 36 SNP markers with significant association with NDF, CAN, CH, GFD, PH, and grain yield. Furthermore, eight markers were significant and common across DI and FI regimes, while 14 markers were uniquely associated with the studied traits under DI. Moreover, under DI and FI, three (11_10326, 11_20042, and 11_20170) and five (11_20099, 11_10326, 11_20840, 12_30298, and 11_20605) markers, respectively, had pleiotropic effects on at least two traits. Among the significant 36 SNP markers, 24 were annotated to known barley genes. Most of these genes were involved in plant responses to environmental stimuli such as drought. Overall, nine of the significant markers were previously reported, and 27 markers might be considered novel. Several markers identified in this study could enable the identification of barley accessions with optimal agronomic performance under DI and FI. Further research is required to verify the novelty of the significant markers across multiple environments.

## Figures and Tables

**Table 1:** The mean performance, coefficient of variance (CV, %) and heritability for the Number of Days to Flowering (NDF), Canopy Temperature (CAN), Chlorophyll Content (CH), Grain-Filling Duration (GFD), Plant Height (PH), and Grain Yield (Yield) for 2019 and 2020 growing seasons under Full Irrigation (FI) and Deficit Irrigation (DI).

Treat	Years	Irrigation	Mean	Standard Error	CV (%)	Heritability within Trials
NDF	2019	Deficit	86.80	1.15	0.93	0.93
		Full	90.92	1.01	1.02	0.89
	2020	Deficit	83.30	0.79	0.88	0.88
		Full	87.32	0.74	0.78	0.89
CAN	2019	Deficit	31.57	1.10	4.81	0.85
		Full	26.32	0.98	5.21	0.77
	2020	Deficit	29.22	0.96	5.53	0.80
		Full	24.30	0.98	5.32	0.83
CH	2019	Deficit	31.57	1.01	5.96	0.77
		Full	26.32	1.17	5.28	0.87
	2020	Deficit	29.22	1.15	8.55	0.77
		Full	24.30	1.37	8.92	0.84
GFD	2019	Deficit	26.27	0.81	4.68	0.92
		Full	30.84	0.80	5.35	0.90
	2020	Deficit	26.34	0.42	5.43	0.86
		Full	31.35	0.41	4.81	0.88
PH	2019	Deficit	103.63	4.81	2.94	0.97
		Full	109.43	5.30	3.67	0.95
	2020	Deficit	90.44	3.39	4.26	0.92
		Full	96.20	3.04	3.34	0.95
Yield	2019	Deficit	3.40	0.40	6.86	0.91
		Full	4.27	0.49	6.66	0.91
	2020	Deficit	3.12	0.26	5.76	0.87
		Full	3.85	0.34	6.33	0.86

**Table 2:** The analysis of variance for the Number of Days to Flowering (NDF), Canopy Temperature (CAN), Total Chlorophyll Content (CH), Grain-Filling Duration (GFD), Plant Height (PH), and Grain Yield (Yield) in 2019 and 2020 under Full Irrigation (FI) and Deficit Irrigation (DI).

Source	df	Mean Square					
		NDF	CAN	CH	GFD	PH	Yield
Years	1	10,841.70	4099.06	49,266.32	70.65	150,070.88	107.74
Iblock (Rep × Years)	38	0.48	1.20	2.92	0.41	13.44	0.09
Stress	1	14,240.12	22,264.68	13.78	19,713.73	28,750.88	553.77
Stress × Years	1	2.74	23.89	40.25	40.88	0.59	3.91
Error A	38	0.52	0.64	2.90	0.23	11.62	0.05
Genotype	429	5.37	3.93	39.69	1.79	145.16	1.16
Genotype × Years	429	1.78	3.45	10.40	1.72	54.34	0.59
Genotype × Stress	429	3.83	3.19	13.14	0.91	23.31	0.19
Genotype × Years × Stress	429	1.19	2.74	10.78	0.87	20.28	0.18
Error	1644	0.37	0.64	3.53	0.28	8.90	0.06
Heritability across trials		0.58	0.20	0.72	0.78	0.94	0.73

## Data Availability

All data reported in this study were provided in the main text or as Supporting Information.

## Supplementary Materials

The following supporting information can be downloaded at: https://www.mdpi.com/article/10.3390/plants11223072/s1, Table S1: Main soil properties for El Khazan location across 2018/2019 and 2019/2020 growing seasons, Table S2: Lsmeans for Number of Days to Flowering (NDF), Canopy Temperature (CAN), Chlorophyll Content (CH), Grain-Filling Duration (GFD), Plant Height (PH), and Grain Yield (GY) for 2019 and 2020 growing seasons under Full Irrigation (FI) and Deficit Irrigation (DI) across the 426 genotypes., Table S3: P-values and effect from the genome wide association scan (GWAS) for the Number of Days to Flowering (NDF), Canopy Temperature (CAN), Total Chlorophyll Content (CH) Grain-Filling Duration (GFD), Plant Height (PH), and Grain Yield (GY) under Full Irrigation (FI) and Deficit Irrigation (DI). Table S4: SNP annotation summary for the significant markers identified for Number of Days to Flowering (NDF), Canopy Temperature (CAN), Chlorophyll Content (CH), Grain-Filling Duration (GFD), Plant Height (PH), and Grain Yield (GY) under Full Irrigation (FI) and Deficit Irrigation (DI).
