# Supplementary material for: Novel Single-Nucleotide Variants for Morpho-Physiological Traits Involved in Enhancing Drought Stress Tolerance in Barley
Source: Plants (Basel). 2022 Nov 13;11(22):3072. doi: 10.3390/plants11223072 (PMC9696095; doi:10.3390/plants11223072)
Supplement: Supplementary file 1 [file plants-11-03072-s001.zip › Table S1.pdf]

TABLE S1. Main soil properties for El Khazan location across 2018/2019 and 2019/2020 growing seasons.

**Soil properties**	2018/2019	2019/2020
Sand (%)	20	20.8
Silt (%)	32.2	32.5
Clay (%)	48.8	46.7
**USDA Texture class**	**Clay**	**Clay**
Water content at FC (%)	40	41
Water content at PWP (%)	21.5	22
AWC (mm m^{-1})	185	190
Organic matter content (%)	0.62	0.63
Bulk Density Mg m^{-3}	1.26	1.21
Total porosity%	53.9	52.8
PH (1:2.5 soil suspension)	8.2	8.3
ECe (ppm)	179	179
CEC (meq/100g)	37	38.14
$CaCO_3$	4.11	3.54

FS, Field capacity

PWP, Permeant wilting point

AWC, Available water capacity

Ece, Electrical conductivity of saturated soil paste extract.

CEC, cation exchange capacity.

OM, organic matter (calculated by multiplying the organic carbon content by a conversion factor of 1.724).
